# Impact of obesity on the short-term outcomes of single-port laparoscopic colectomy for colorectal cancer in the Asian population: A retrospective cohort study

**DOI:** 10.1097/MD.0000000000006649

**Published:** 2017-07-14

**Authors:** Byung Jo Choi, Won Jun Jeong, Say-June Kim, Sang Chul Lee

**Affiliations:** Department of Surgery, College of Medicine, Daejeon St. Mary's Hospital, The Catholic University of Korea, Daejeon, Republic of Korea.

**Keywords:** Asian, colorectal cancer, obesity, single-port laparoscopic surgery

## Abstract

Single-port laparoscopic surgery (SPLS) is being increasingly performed for treating colorectal cancer. Here, we aimed to assess the safety and feasibility of SPLS for colorectal cancer in obese patients through a comparison of their short-term outcomes with those of nonobese patients.

A total of 323 patients who underwent SPLS for colorectal cancer at our center between March 2009 and August 2014 were enrolled. The outcomes were analyzed according to the body mass index (BMI) category: nonobese (BMI < 25), obese I (BMI: 25.0–29.9), and obese II (BMI ≥ 30).

Of the 323 patients, 233 (72.1%), 80 (24.8%), and 10 (3.1%), were assigned to the nonobese, obese I, and obese II groups, respectively. The clinicopathologic patient characteristics, such as age, gender, tumor location, and previous laparotomy, were similar among the 3 groups. The mean operative time (nonobese vs obese I vs and obese II groups: 269.2 vs 270.4 vs 342.8 minutes, respectively) and estimated surgical blood loss (277.7 vs 260.5 vs 387.0 mL, respectively) were greater in the obese II group than in the nonobese and obese I groups, although the difference was not significant (*P* = .247 and *P* = .205, respectively). However, the time to passage of flatus significantly differed among the groups (*P* = .040); in particular, this value was significantly longer in the obese II group than in the obese I group (*P* = .031). None of the other parameters, including conversion to open or conventional laparoscopic surgery and intra- and postoperative morbidity, significantly differed among the 3 groups.

SPLS for colorectal cancer can be safely performed in obese Asian patients with equivalent short-term outcomes as compared with that in nonobese patients. Hence, SPLS can be safely recommended for colorectal cancer in obese patients if the surgeon is experienced. Nevertheless, the technique used warrants further investigation, and a large-scale prospective study is required.

## 1 Introduction

Laparoscopic resection of colorectal cancer has been proven to be safe, and is associated with less postoperative pain, earlier recovery, and similar cancer survival as compared with traditional open surgery.^[1,2]^ However, laparoscopic colectomy involves a longer operation time and greater learning curve. Several factors, including patient- and disease-related factors, prior abdominal surgery, tumor bulk, and disease location, can affect the success of laparoscopic surgery. Of these factors, obesity—defined as a body mass index (BMI) of >30 kg/m^2^—is considered a contraindication to the laparoscopic approach.^[3,4]^ Obesity is associated with many comorbidities, such as diabetes, hypertension, and cardiovascular disease.

Moreover, abdominal surgery in obese patients is technically difficult because of the presence of bulky fatty tissue and a vague dissection line as a result of a heavy and thick mesentery. Thus, obesity is considered to increase the postoperative complication rate. However, once the surgeon gains sufficient experience, obesity does not pose a challenge for laparoscopic colectomy. Several reports have described the safety and feasibility of laparoscopic colectomy in obese patients. Thus, the findings of laparoscopic colectomy are controversial. Some studies suggest that laparoscopic colectomy can be safely performed in obese patients,^[5,6]^ whereas some other studies state that obesity has a negative impact on operative time, postoperative complications, and conversion rate.^[7,8]^

Single-port laparoscopic surgery (SPLS) is an advanced type of conventional laparoscopic surgery. In recent years, SPLS—particularly single-port laparoscopic colorectal surgery—has been constantly evolving. Various studies have reported on single-port laparoscopic colorectal surgery, and several reports have demonstrated that SPLS for colorectal cancer has similar short-term outcomes as compared with conventional laparoscopic surgery.^[9,10]^ However, no report on the safety of SPLS for colorectal cancer among obese patients has been published thus far. In some previous reports, SPLS colectomy was primarily performed in patients without any prior abdominopelvic surgeries and a BMI of <30 kg/m^2^ to avoid technical difficulty and unfamiliarity with altered anatomy.^[11,12]^ In the Asian population, the frequency of obesity and BMI is lower, and the amount of fat deposits is higher than in the Western population.^[13]^ It may be technically challenging to perform the laparoscopic technique including SPLS in such cases of visceral obesity. In the present study, we aimed to assess the safety and feasibility of SPLS for colorectal cancer in obese patients by comparing their short-term outcomes with those of nonobese patients.

## 2 Methods

From March 2009 to August 2014, a total of 323 consecutive patients underwent SPLS for colorectal cancer at Daejeon St. Mary Hospital, which is affiliated with The Catholic University of Korea. The inclusion criteria for patients eligible for SPLS were: age ≥18 years and a clinical diagnosis of adenocarcinoma or other malignancy originating in the colon and/or rectum. All patients who were eligible for conventional laparoscopic procedures met the inclusion criteria. A specific BMI cutoff and previous abdominal surgery history were not considered exclusion criteria. The prospectively collected data from the 323 patients were retrospectively reviewed. Patients were excluded if they had undergone emergency SPLS or if their surgery had included the combined resection of other organs. The study was approved by the ethics committee of Daejeon St. Mary's hospital, the Catholic University of Korea (IRB code: DC16RISI0050). The protocol for single-port laparoscopic colorectal surgery has been standardized, as reported previously.^[14,15]^ All surgeries were performed by 1 expert surgeon (SCL).

The BMI (calculated using height and body weight; kg/m^2^) was used as an objective measure of obesity. In accordance with the WHO classification of obesity in the Asia-Pacific region,^[16]^ patients were classified into 3 groups: nonobese (BMI < 25.0 kg/m^2^), obese I (BMI: 25.0–29.9 kg/m^2^), and obese II (BMI ≥ 30.0 kg/m^2^).

The patient characteristics including age, sex, BMI, American Society of Anesthesiologists (ASA) score, comorbidity, previous abdominal surgery, and tumor location; surgery-related parameters including operative time, blood loss, conversion to multiport or open surgery, length of hospital stay, time to first flatus, time to first feeding, and intraoperative complications; pathologic parameters including tumor stage and number of harvested lymph nodes; and postoperative parameters including complications, 30-day reoperation, and 30-day mortality were assessed.

Complications were defined as the presence of any adverse events that developed perioperatively or within 30 days after the procedure. Conversion was defined as the completion of any part of a procedure by using an open technique, except for specimen delivery or the construction of an intestinal anastomosis. Intestinal obstruction was defined as the inability to tolerate a solid diet with concomitant radiologic findings suggestive of total mechanical obstruction of the large or small intestine. Urinary retention was defined as a need for reinsertion of the urinary catheter to pass urine after urinary catheter removal.

Data are presented as the number of patients and percentage, or as the mean and standard deviation. Continuous data were analyzed using the Kruskal–Wallis test. Moreover, Fisher exact test or *χ*^2^ test was used to compare categorical data. A 2-sided *P* value of <.05 was considered significant. All data analyses were performed using SPSS version 17 (SPSS Inc, Chicago, IL).

## 3 Results

The patient characteristics are shown in Table 1. Of the 323 patients, 233 (72.1%), 80 (24.8%), and 9 (3.1%), were assigned to the nonobese, obese I, and obese II groups, respectively. The clinicopathologic patient characteristics, such as age, gender, tumor location, and previous laparotomy history, were similar among the 3 groups. The operative procedures did not significantly differ among the groups, although the rate of anterior resection was higher in the obese II group than in the nonobese and obese I groups (50% vs 27.9% and 23.8%, respectively). The operative and postoperative outcomes are shown in Table 2. There were no significant differences among the 3 groups in terms of mean operative time (269.2 vs 270.4 vs 342.8 min, *P* = .247) and mean blood loss (277.7 vs 260.5 vs 387.0 mL, *P* = .205). However, the time to the passage of flatus significantly differed among the 3 groups (*P* = .040); this value was significantly longer in the obese II group than in the obese I group (*P* = .031) but not in the nonobese group (*P* = .066). No other significant differences, including conversion to open or conventional laparoscopic surgery and intra- and postoperative morbidity, were found among the 3 groups. Conversion to open colectomy was noted in 2 and 1 cases in the nonobese group and the obese I group, respectively, due to dense adhesions in 1 case and massive bleeding in 2 cases; however, there were no cases of conversion to conventional multiport laparoscopic surgery. Two cases of death due to myocardial infarction and sepsis were noted in the nonobese group, but no cases of death were noted in the obese I and obese II groups. The pathologic outcomes are shown in Table 3. There were no significant differences among the 3 groups in terms of pathologic data, including the mean number of harvested lymph nodes.

**Table 1:**
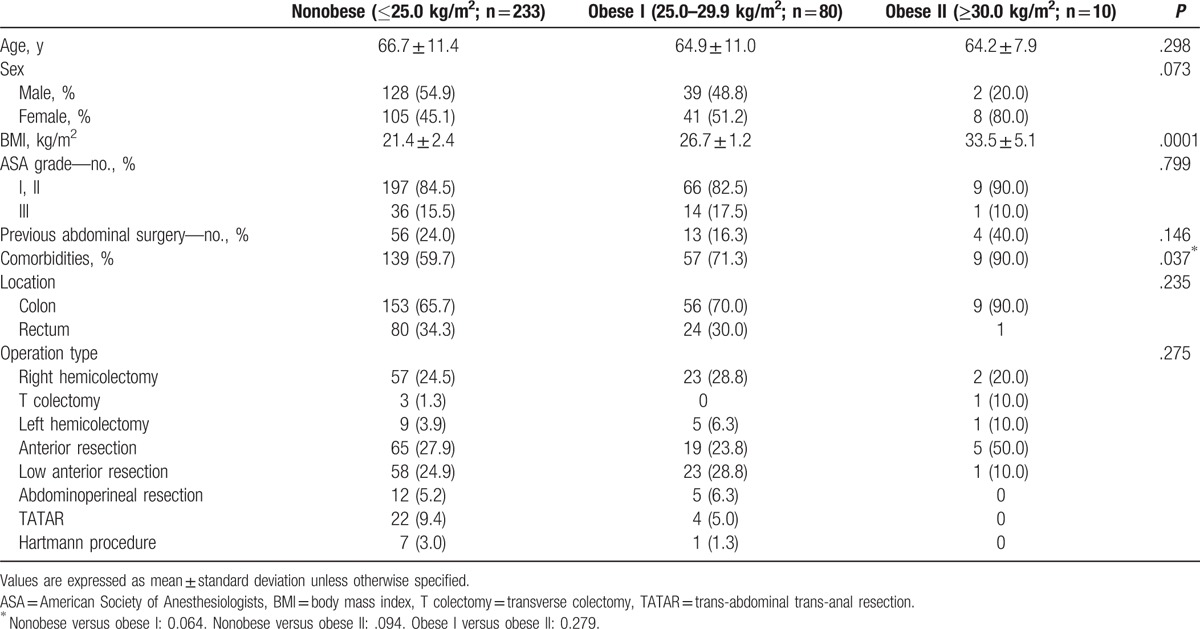
Clinicopathologic patient characteristics.

**Table 2:**
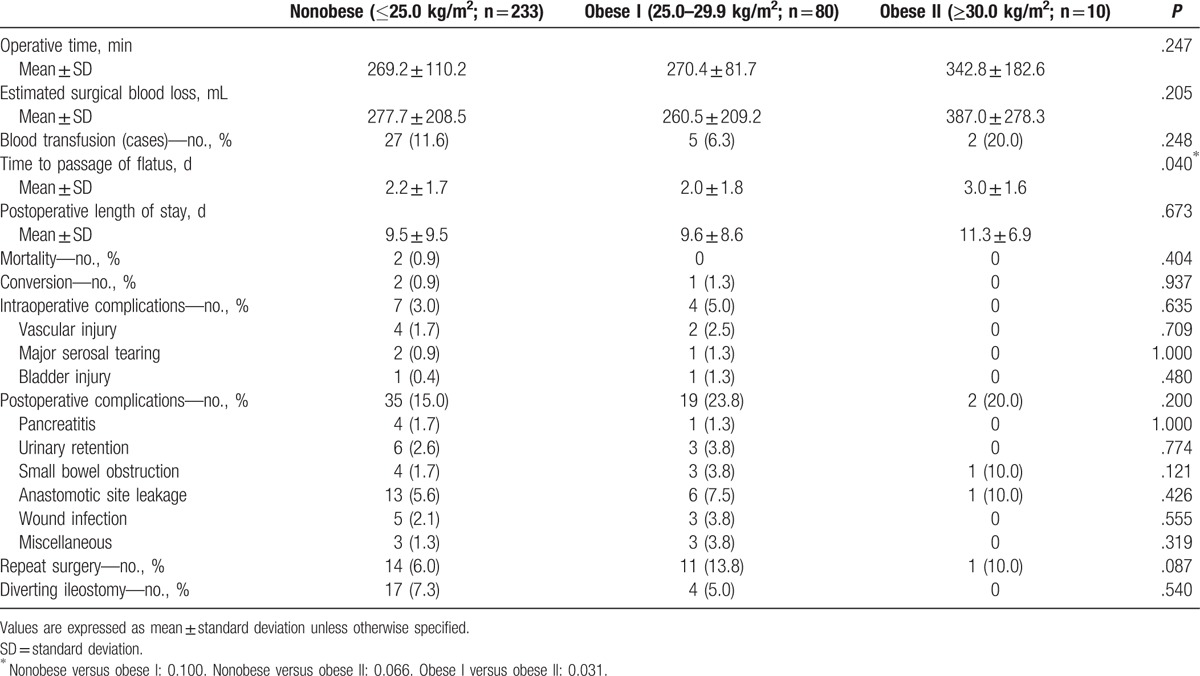
Operative and postoperative data.

**Table 3:**
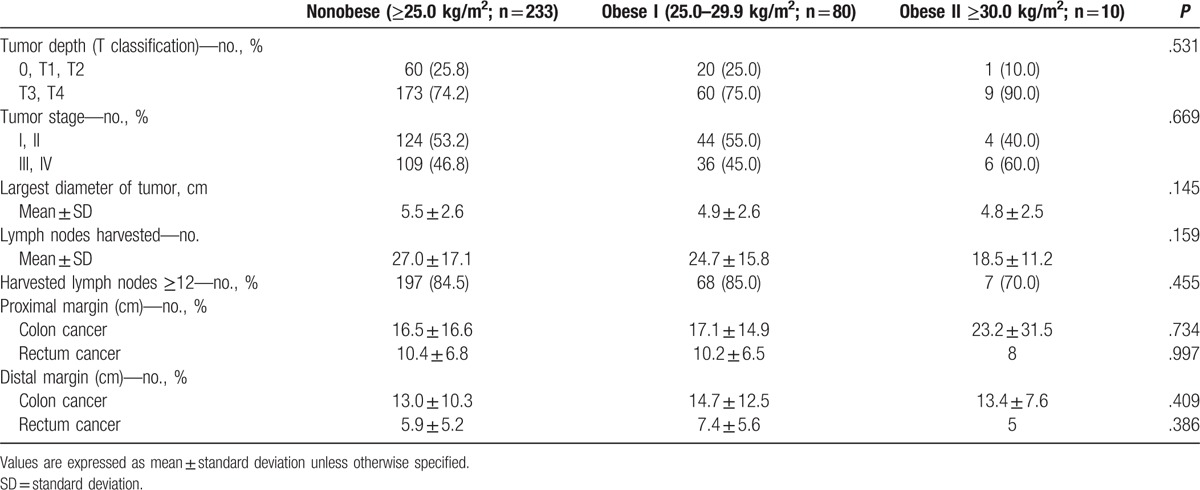
Data related to tumor pathology in patients undergoing single-port colectomy.

## 4 Discussion

Although the laparoscopic technique is commonly adopted in colorectal surgery, the impact of obesity on the outcomes of laparoscopic colectomy remains controversial. The increase in the prevalence of obesity and its accompanying comorbidities are major concerns in industrialized countries. Approximately 33.8% of adult Americans are estimated to be obese, with a BMI of >30.^[17]^ Several studies on obesity and laparoscopic surgery have been published thus far, and the findings differ based on the center. However, in general, the average BMI of Asian individuals is lower than that of non-Asian communities.^[18]^ Therefore, only a few studies have been conducted in Asian countries, as compared with Western countries. Obesity is defined as a body mass index of ≥25 kg/m^2^ in Korea. At present, 32.8% of adults (36.1% of men and 29.7% of women) are reported to be obese in Korea.^[19]^ Asian individuals generally have a higher body fat percentage as compared with individuals from Western countries at the same BMI level.^[20]^ The increased levels of fat tissue in obese patients affect surgery by inhibiting the visualization, dissection of the tissue planes, and ligation of the vessels.^[21]^ SPLS, a minimally invasive technique that maximizes the benefits of laparoscopic surgery, has been constantly evolving, particularly in colorectal surgery. We evaluated the impact of obesity after SPLS for colorectal cancer by using a BMI of 25 kg/m^2^ as a cut-off value for nonobese and obese patients (obese I and II, according to a BMI of 30 kg/m^2^).

Since the first single-port colectomy was performed in our institution in March 2009, SPLS for colorectal cancer has been routinely conducted as a minimally invasive surgery. Thereafter, several advancements in the surgical procedure have been made and the protocol for SPLS has been standardized. Our previous studies at the institute on the short-term outcomes between SPLS and conventional laparoscopic surgery for colorectal cancer indicated the feasibility and safety of SPLS for colorectal cancer.^[14,15]^ In the present study, we found that obese patients with higher BMI had comparable short-term outcomes of SPLS for colorectal cancer with individuals with a normal weight.

Previous studies on the surgical outcome of conventional laparoscopic colectomy for colorectal cancer in obese patients have reported various results; some studies suggested that it was safe and feasible,^[22–24]^ whereas others indicated that it had more frequent postoperative complications,^[25]^ longer operation times,^[25,26]^ increased blood loss,^[27]^ and increased conversion rates.^[28]^ A study of the early postoperative complications showed that the frequency of postoperative complications after laparoscopic colorectal surgery was not greater among obese patients than among nonobese patients.^[24]^

In a review and meta-analysis of 33 studies, Makino et al^[29]^ evaluated the feasibility and safety of laparoscopic colectomy for colorectal diseases between obese patients and nonobese patients. In the present study, we did not observe any evidence of a negative impact of obesity on intraoperative blood loss, perioperative mortality, and reoperation rate. However, it is unclear whether obesity is a risk factor for wound infection after laparoscopic colectomy.^[29]^ With regard to the long-term oncologic outcomes, Makino et al^[26]^ performed a case-matched, comparative study on 152 patients, and found that laparoscopic colonic resection is safe and reasonable in obese patients; in fact, this procedure was shown to yield similar short-term and oncologic outcomes in obese patients as in nonobese patients. However, the inclusion criteria for the patients are varied among the different studies; hence, it difficult to draw any definitive conclusions. Nevertheless, the studies from Western countries generally suggest that laparoscopic colorectal surgery in obese patients with a BMI of ≥30 kg/m^2^ is feasible, but technically demanding.

As mentioned above, only a few studies have examined the impact of obesity on the outcomes of conventional laparoscopic colorectal surgery in Asian populations, as a result of the smaller obese population as compared with that in Western countries. In the present study, 24.8% of cases had a BMI between 25.0 and 29.9 and 3.1% had a BMI of ≥30.0 kg/m^2^, consistent with the ratios of other Asian studies.^[30,31]^

Previous studies in Asian countries have assessed the influence of obesity on the surgical outcomes of conventional laparoscopic colorectal surgery; those studies have primarily used BMI of ≥25.0 kg/m^2^ as a cutoff for obesity. Two large retrospective studies of 1194 Japanese and 984 Korean patients reported that obesity II—defined as BMI of ≥30 kg/m^2^—had a significant negative impact on the mean operating time and mean estimated blood loss. However, in the obesity I group (defined as a BMI between 25.0 and 30 kg/m^2^), 1 study showed a greater operating time and estimated blood loss as compared with nonobese patients, whereas another study showed similar findings without any significant differences. With regard to morbidity, the rate of postoperative complications was significantly higher in obese II patients than in nonobese and obese I patients (24% vs 9.2% vs 9.1%, *P* = .0428) in the former study of Japanese patients, although the rates of intraoperative events (*P* = .634) and postoperative complications (*P* = .603) were similar between nonobese and obese patients in the study of Korean patients.^[30,31]^

In the present study, the short-term outcomes of single-port laparoscopic colorectal surgery in obese patients were comparable to those in nonobese patients. There were no significant differences among the 3 groups in terms of the operating time, estimated blood loss, postoperative complications, conversion rate, and blood loss. There was a significant difference in the time to passage of flatus among the 3 groups. However, the recovery from gastrointestinal surgery depends on the extent of the operation, location of the colonic resection, and creation of stoma. When the data of patients with colonic resection without stoma were analyzed, we did not find any significant differences among the 3 groups in terms of the time to passage of flatus (2.16 vs 2.21 vs 2.89 days, *P* = .167). The operating time and estimated blood loss tended to be higher in the obese II group. Nevertheless, the sample size of obese II patients (10 patients, 3.1%) was very small in the present study. As this fact may have affected the study findings, we believe that this represents a limitation of this study. Another study has analyzed the influence of obesity on the short-term outcomes of laparoscopic colectomy for colorectal cancer by using a BMI cut-off value of 28 kg/m^2^ in the Asian population to avoid such limitation. In that study, no significant differences in operative time, blood loss, intraoperative complications, and conversion rates were noted. The postoperative complications and duration of postoperative hospital stay were also similar among the 3 groups.^[23]^ In a comparative study of open colectomy in 166 obese Chinese patients (defined as BMI of ≥ 28 kg/m^2^), the authors noted improved short-term outcomes, in terms of operating time, estimated blood loss, and wound infection, in laparoscopic colectomy for colorectal cancer patients with a BMI of >28, as compared with patients who underwent an open procedure.^[32]^

The single-port laparoscopic colorectal surgery in the high BMI group was technically demanding due to the problems of surgical field exposure and difficulties in dissection. An aggressive position change of the patient was needed and an intraperitoneal gauze was used intraoperatively to secure the surgical field. However, this process was not sufficiently time-consuming to lead to a significantly increased operative time and conversion rate. With regard to the pathological outcomes, the number of lymph nodes harvested and the margins and lengths of the resected specimens were adequate among the 3 groups. These results suggest that single-port laparoscopic colorectal surgery, when performed by an experienced laparoscopic surgeon, has similar outcomes in patients with high BMI.

We acknowledge that this study has several limitations, including the small sample size, lack of long-term oncologic outcomes, and retrospective nature of the study. Furthermore, a selection bias for surgical candidates and the proficiency of the SPLS operator could be other limitations of the study. Nevertheless, in this study, we performed single-port laparoscopic colorectal surgery in consecutive patients with colorectal cancer. In addition, during patient selection, we did not exclude obese patients or those with a history of abdominal surgery. Patients who were eligible for conventional laparoscopic procedures met the inclusion criteria for SPLS.^[14,15]^ To our knowledge, this is the first report to analyze the impact of obesity on the short-term outcomes after single-port laparoscopic colorectal surgery. Thus, these data will be useful for experienced surgeons who decide to perform SPLS for obese patients with colorectal cancer.

In the present study, we found that the operating time, blood loss, postoperative hospital stay, perioperative complications, and conversion rate did not significantly differ among morbidly obese, obese, and nonobese Asian individuals who underwent SPLS for colorectal cancer. Thus, SPLS for colorectal cancer can be safely conducted in obese patients, when performed by an experienced SPLS surgeon, with beneficial short-term outcomes, similar to those of conventional laparoscopic surgery. Nevertheless, further investigations and large-scale prospective studies are needed to validate this finding.
